# Sex differences in the association of Alzheimer’s disease biomarkers and cognition in a multicenter memory clinic study

**DOI:** 10.1186/s13195-025-01684-z

**Published:** 2025-02-18

**Authors:** Cecilia Boccalini, Debora Elisa Peretti, Max Scheffler, Linjing Mu, Alessandra Griffa, Nathalie Testart, Gilles Allali, John O. Prior, Nicholas J. Ashton, Henrik Zetterberg, Kaj Blennow, Giovanni B. Frisoni, Valentina Garibotto

**Affiliations:** 1 Laboratory of Neuroimaging and Innovative Molecular Tracers (NIMTlab), Faculty of Medicine, Geneva University Neurocenter, University of Geneva, Rue Gabrielle-Perret-Gentil 4, Geneva, CH-1205 Switzerland; 2 Division of Radiology, Geneva University Hospitals, Rue Gabrielle-Perret-Gentil 4, Geneva, CH-1205 Switzerland; 3 Institute of Pharmaceutical Sciences, ETH Zurich, Vladimir-Prelog-Weg 1-5/10, Zurich, 8049 Switzerland; 4 Leenaards Memory Center, Department of Clinical Neurosciences, Lausanne University Hospital and University of Lausanne, Chem. de Mont-Paisible 16, Lausanne, 1011 Switzerland; 5 Medical Image Processing Laboratory, Neuro-X Institute, École Polytechnique Fédérale De Lausanne– EPFL, Campus Biotech H4 Chemin des Mines 9, Geneva, CH-1202 Switzerland; 6 Nuclear Medicine and Molecular Imaging, Lausanne University Hospital, Rue du Bugnon 46, Lausanne, 1005 Switzerland; 7 Centre for Age-Related Medicine, Stavanger University Hospital, Armauer Hansens vei 30, Stavanger, 4011 Norway; 8 Department of Psychiatry and Neurochemistry, Institute of Neuroscience and Physiology, The Sahlgrenska Academy at the University of Gothenburg, Wallinsgatan 6, Mölndal, S-431 80 Sweden; 9 Institute of Psychiatry, Psychology & Neuroscience, King’s College London, Maurice Wohl Clinical Neuroscience Institute, London, SE5 9RX UK; 10 UK NIHR Biomedical Research Centre for Mental Health & Biomedical Research Unit for Dementia at South London & Maudsley NHS Foundation, London, SE5 8AF UK; 11 UK Dementia Research Institute at UCL, London, WC1E 6BT UK; 12 UK Department of Neurodegenerative Disease, UCL Institute of Neurology, London, WC1N 3BG UK; 13 Clinical Neurochemistry Laboratory, Sahlgrenska University Hospital, Klin Neurokemi Lab Hus V3, SU/Mölndals sjukhus, Mölndal S-431 80, Gothenburg, Sweden; 14 Hong Kong Centre for Neurodegenerative Diseases, Clear Water Bay, Hong Kong Units, Hong Kong, 1501-1502, 1512-1518 China; 15 Wisconsin Alzheimer’s Disease Research Centre, University of Wisconsin, School of Medicine and Public Health, University of Wisconsin-Madison, Madison, WI 53792 USA; 16 Pitié Salpêtrière Hospital, Paris Brain Institute, ICM, Sorbonne University, 47 Bd de l’Hôpital, Paris, 75013 France; 17 Neurodegenerative Disorder Research Centre, Division of Life Sciences and Medicine, Department of Neurology, Institute on Aging and Brain Disorders, University of Science and Technology of China and First Affiliated Hospital of USTC, Hefei, 230001 China; 18 Geneva Memory Center, Geneva University Hospitals, Rue Gabrielle-Perret-Gentil 4, Geneva, CH-1205 Switzerland; 19 Division of Nuclear Medicine and Molecular Imaging, Geneva University Hospitals, Rue Gabrielle-Perret-Gentil 4, Geneva, CH-1205 Switzerland; 20 CIBM Center for Biomedical Imaging, EPFL AVP CP CIBM Station 6, Lausanne, 1015 Switzerland

**Keywords:** Sex, Neuroimaging, Alzheimer’s disease, Biomarkers, tau-PET, Women

## Abstract

**Background:**

This study investigated sex differences in the associations between Alzheimer’s disease (AD) biomarkers, cognitive performance, and decline in memory clinic settings.

**Methods:**

249 participants (females/males:123/126), who underwent tau-PET, amyloid-PET, structural MRI, and plasma glial fibrillary acidic protein (GFAP) measurement were included from Geneva and Lausanne Memory Clinics. Mann-Whitney U tests investigated sex differences in clinical and biomarker data. Linear regression models estimated the moderating effect of sex on the relationship between biomarkers and cognitive performance and decline. Sex differences in cognitive decline were further evaluated using longitudinal linear mixed-effect models with three-way interaction effects.

**Results:**

Women and men present similar clinical features, amyloid, and neurodegeneration. Women had higher tau load and plasma levels of GFAP than men (*p* < 0.05). Tau associations with amyloid (standardized β = 0.54,*p* < 0.001), neurodegeneration (standardized β=-0.44,*p* < 0.001), and cognition (standardized β=-0.48,*p* < 0.001) were moderated by a significant interaction with sex. Specifically, the association between amyloid and tau was stronger among women than men (standardized β=-0.19,*p* = 0.047), whereas the associations between tau and cognition and between tau and neurodegeneration were stronger among men than in women (standardized β=-0.76,*p* = 0.001 and standardized β=-0.56,*p* = 0.044). Women exhibited faster cognitive decline than men in the presence of severe cortical thinning (*p* < 0.001).

**Conclusion:**

Women showed higher tau load and stronger association between amyloid and tau than men. In individuals with high tau burden, men exhibited greater neurodegeneration and cognitive impairment than women. These findings support that sex differences may impact tau deposition through an upstream interplay with amyloid, leading to downstream effects on neurodegeneration and cognitive outcomes.

**Supplementary Information:**

The online version contains supplementary material available at 10.1186/s13195-025-01684-z.

## Background

The prevalence of Alzheimer’s disease (AD) continues to rise with women having a greater lifetime risk of developing AD (1 in 5) compared with men (1 in 10) [1]. Higher longevity generally experienced by women is not a sufficient explanation for sex differences, which instead also contribute to disease-related pathophysiological changes [2]. Sex differences likely arise from both distinct and interacting effects of gonadal hormones and sex chromosomes on neuroinflammation, epigenetics, metabolism, autophagy, and other molecular processes [3]. Indeed, sex has been shown to modulate risk factors and potential disease-causing mechanisms in AD and other neurodegenerative diseases.

According to the amyloid cascade hypothesis [4], amyloid deposition in the cortex represents the first event and its association with elevated tau deposition in medial temporal lobes facilitates tau spread to neocortical areas. This combination likely leads to neurodegeneration which in turn exacerbates cognitive impairment and dementia. Meanwhile, a complex array of molecular and cellular network changes could also occur in the brain, including neuroinflammation, synaptic dysfunction, and vascular injury [5] that are not specific to AD but still important in its pathogenic pathway [6]. These pathophysiological mechanisms are characterized by wide intersubject heterogeneity [7], and many of them exhibit variations based on sex [3]. Neuropathological studies across samples ranging from normal cognition to dementia have found that females exhibit a greater burden of AD neuropathology, specifically neurofibrillary tau tangles, compared to males [8]. In line with postmortem findings [8], a greater burden and a faster accumulation rate of tau tangles in females than males was reported in vivo in both symptomatic and presymptomatic individuals using Positron Emission Tomography (PET) [9–17]. A faster tau accumulation in females than males seemed to be facilitated by sex-specific modulation of cortical amyloid on tau phosphorylation [12], and, interestingly, a greater effect of amyloid on tau phosphorylation has been found in the presence of astrocyte reactivity measured by plasma glial fibrillary acidic protein (GFAP) in men than in women [18], even if preliminary results showed higher GFAP levels in females [19].

Despite the growing recognition of sex differences in AD pathology burden and accumulation over time, sex differences in the wide range of biological mechanisms underpinning AD and the complex interplay leading to cognitive impairment and decline require further investigation. This study aims to investigate sex differences in pathological protein deposition, comprehensively considering other important biomarkers in the AD pathogenic pathway, such as neurodegeneration, neuroinflammation, and vascular brain injury [6], and their impact on cognition.

Firstly, we assessed sex differences in clinical variables and biomarker data, including amyloid and tau measured by PET, neurodegeneration measured by magnetic resonance imaging (MRI) cortical thickness and early-phase tau-PET perfusion, astrocytic reactivity as neuroinflammation marker measured by plasma GFAP, and vascular brain injury, in a memory clinic setting. Secondly, we investigated whether the specific relationships between biomarkers, following the AD temporal sequence [4], differ by sex. Lastly, sex moderation effects were also assessed in the associations between biomarkers and cognitive performance and decline.

## Methods

### Participants

The study included subjects assessed at the Geneva Memory Clinic (Geneva University Hospitals) and the Leenaards Memory Center (Lausanne University Hospital), ranging from cognitively unimpaired (CU) to mild cognitive impairment (MCI) and dementia. The local ethics committee approved the different imaging studies, which were conducted under the principles of the Declaration of Helsinki and the International Conference on Harmonization Good Clinical Practice. All participants signed an informed consent to participate in the study.

We included a total of 249 subjects (females/males: 123/126) classified as CU (*n* = 66), MCI (*n* = 127), and dementia (*n* = 56) subjects, following standardized criteria for clinical staging [20–22]. Inclusion criteria were at least one tau-PET scan using ^18^F-flortaucipir, a 3-dimensional T1-weighted MRI scan, a Mini-Mental State Examination (MMSE), and an interval of less than 1 year between measures. A subsample of 186 underwent an amyloid-PET within 1 year. A subsample of 171 participants underwent dual-phase protocol for tau-PET allowing us to have a measure of perfusion as a surrogate of neurodegeneration. *APOE* genotyping has been performed for a subgroup of 152 participants (females/males: 78/74). Plasma samples for GFAP as a measure of neuroinflammation were available for 135 subjects (females/males: 67/68) (see below and Table S1 for subsample’s features). At least one clinical follow-up was available for 137 individuals (46 CU, 73 MCI, and 18 patients with dementia) at 26.68 ± 12.82 months (Table S2 for subsample’s features). Two follow-ups were available for 37 out of 137 and 12 of them also had a third one.

### Imaging acquisition and preprocessing

**MRI -** High-resolution anatomical 3D T1 MRI images were obtained at Geneva University Hospitals’ Division of Radiology on a 3 Tesla scanner (Magnetom Skyra, Siemens Healthineers, Erlangen) using a T1-weighted Magnetization-Prepared Rapid Acquisition Gradient Echo (MPRAGE) protocol with TR 1810 ms, TI 900 ms, TE 2.19 ms, flip angle 8◦, matrix size of 256 × 256, in-plane resolution of 1.0 × 1.0 mm2, slice thickness of 1 mm; and Lausanne University Hospital on Siemens 3 Tesla scanners (Magnetom Prismafit, Skyra, Vida, Siemens Healthineers, Erlangen, Germany) using an MPRAGE protocol with TR 2300 ms, TI 900 ms, TE 2.98 ms, flip angle 9◦, matrix size of 256 × 256, in-plane resolution of 1.0 × 1.0 mm^2^, slice thickness 1.1 mm following the ADNI MR protocol guidelines. White matter lesions were visually rated with the age-related white matter change scale (ARWMC) [23]. The lesion prediction algorithm [24], implemented in the lesion segmentation toolbox for Statistical Parametric Mapping (SPM) software package running in MATLAB, was used to segment fluid-attenuated inversion recovery images, allowing us to extract the total lesion volume (TLV). T1 MRI images were segmented, and volumes and cortical thickness were extracted using FreeSurfer (v.7.0; surfer.nmr.mgh. harvard.edu/), resulting in native space parcellations of each participant’s brain using the Desikan–Killiany atlas [25]. Hippocampal volume (HPV) was extracted and adjusted for intracranial volume. An AD cortical signature (weighted average cortical thickness in the entorhinal, inferior temporal, middle temporal, and fusiform regions of interest (ROIs)) was created [26].

**PET -** PET scans were performed at the Division of Nuclear Medicine and Molecular Imaging at Geneva University Hospitals and Lausanne University with Biograph128 mCT, Biograph128 Vision 600 Edge, Biograph 64 Vision 600, Biograph40 mCT, or Biograph64 TruePoint PET-CT scanners (Siemens Healthineers, Erlangen, Germany and Siemens Medical Solutions, Malvern, PA, USA) as well as Discovery D690 TOF (GE HealthCare, Waukesha, WI). All scanners were harmonized regarding their performance and reconstructions, with cross-calibration. [^18^F]flortaucipir (FTP) was synthesized at the Center for Radiopharmaceutical Sciences in ETH Zurich, Switzerland, under license from the intellectual property owner (Avid subsidiary of Lilly, Philadelphia, PA, USA), and used for tau-PET scans. Subjects received 197 ± 39 MBq of FTP, with early-phase static image acquisition started immediately after tracer injection (acquisition time 10 min for Geneva and 6 min for Lausanne) and a late standard acquisition performed 75 min after injection (acquisition time 30 min) [27]. Each emission frame was reconstructed in 6 × 5 min frames and then averaged into a single image. Amyloid-PET images were acquired at Geneva University Hospitals using either [^18^F]florbetapir (FBP) or [^18^F]flutametamol (FMM). In the case of FBP, PET scans were conducted 50 min after the intravenous administration of 210 ± 18MBq of FBP, consisting of 3 × 5 min image frames. For FMM, images were acquired 90 min after the intravenous administration of 166 ± 16MBq of FMM, involving 4 × 5 min image frames. Subsequently, PET images were averaged to create a single frame lasting either 15 (FBP) or 20 (FMM) minutes. For all tracers, data were acquired in list mode and reconstructed using 3D OSEM (Ordered Subset Expectation Maximization). The reconstruction process involved corrections for randoms, dead time, normalization, scatter, attenuation, and sensitivity. After applying motion correction, a 2 mm Gaussian filter with a full width at half maximum (FWHM) was employed. The resulting images had a matrix size of 400 × 400 and isotropic voxels measuring 1.01 mm.

Amyloid-PET data preprocessing procedures and Centiloid calculation have been previously described [28]. A Centiloid value of 19 was used as the cut-off point for amyloid status. For tau-PET preprocessing, each participant’s mean PET image underwent rigid coregistration to its respective native T1-weighted MRI image, and images were intensity-normalized using an inferior cerebellar gray matter reference region, resulting in standardized uptake value ratios (SUVR) images. FreeSurfer parcellations were used to extract mean SUVR within different ROIs for each participant in the native space using PetSurfer. A global tau SUVR was calculated from the entorhinal cortex, lateral occipital cortex, inferior temporal cortex, and amygdala [29], constituting the meta-ROI, and in Braak regions (Braak I-II: hippocampus; Braak III: parahippocampus gyrus, lingual gyrus, amygdala; Braak IV: inferior temporal cortex, middle temporal cortex, temporal pole, thalamus, posterior cingulate, insula; Braak V: frontal cortex, parietal cortex, occipital cortex, superior temporal cortex precuneus, caudate nucleus, putamen; Braak VI: precentral gyrus, postcentral gyrus, paracentral gyrus, cuneus). Early-phase tau-PET processing was performed as previously described [30, 31] using SPM 12, running in MATLAB R2018b, version 9.5 (MathWorks Inc.). Early-phase SUVR images were calculated by normalizing the uptake to the mean value of the pons and cerebellar vermis together as the reference region. Intensity-normalized PET images were saved and entered in a voxel-wise linear regression model in SPM12 with the MMSE to identify an AD-related ROI. The statistical threshold was set at *p* < 0.005, family-wise error (fwe)-corrected at the cluster level. The obtained AD-related ROI resembled the AD-typical hypometabolic pattern including temporoparietal and frontal regions (Figure S1) and has been used to extract the SUVR for early-phase perfusion images.

### Plasma sampling and processing

Plasma samples were available for 135 subjects (females/males: 67/68) (Table S1) and were collected within a year of tau-PET examination, with participants non-fasting. Blood was collected in EDTA-plasma tubes and centrifuged (2000*g*, +4^o^C for 10 min). Following centrifugation, plasma was aliquoted into 1.5 ml polypropylene tubes (1 ml plasma in each tube) and stored at -80^o^C in polypropylene tubes. GFAP concentration was measured using GFAP Simoa Discovery kits for HD-X (Quanterix, Billerica, MA).

### Statistical analysis

Mann-Whitney U tests were performed to explore sex differences in age, years of education, MMSE, and AD biomarkers between groups. Table 1 shows the full panel of biomarkers and related pathological processes. MANCOVA tests were further used to assess differences in each biomarker taking into consideration age and the remaining biomarkers as covariates. A chi-square test was used to compare diagnostic stages, amyloid positivity, and *APOE* carriership between the groups.

**Table 1:** Panel of biomarkers with related pathological processes

Biomarker	Measure	Pathological process
Amyloid-PET	Centiloid	Amyloid deposition
Tau-PET	Tau SUVR in the MetaROI Tau SUVR in Braak stages	Tau deposition
Early-phase tau-PET	Early-phase tau SUVR in AD-related ROI	Perfusion as a proxy of neurodegeneration
T1 MRI	AD cortical thickness Hippocampal volume	Neurodegeneration
GFAP	GFAP **blood** level	Astrocytic reactivity as proxy of neuroinflammation
FLAIR MRI	ARWMC TLV	White matter lesions as cerebrovascular measures

Abbreviations: AD, Alzheimer’s disease; ARWMC, age-related white matter change scale; FLAIR, Fluid attenuated inversion recovery; GFAP, Glial Fibrillary Acidic Protein; MRI, Magnetic resonance imaging; PET, positron emission tomography; ROI, region of interest; SUVR, standardized uptake value ratios; TLV, total lesion volume.

First, separate linear regression models were performed to assess the correlation between AD biomarkers, MMSE, and MMSE rate of changes (calculated by subtracting the MMSE score at baseline from the last follow-up MMSE score and then dividing the result by the number of years of follow-up, thus expressing the average number of MMSE points lost per year) in the whole sample and separately in females and males. Then, a series of separate general linear models including interaction terms were examined to estimate the moderating effect of sex on the relationship between biomarkers, MMSE at baseline, and MMSE annual rate of change. Associations within AD biomarkers were tested based on a biomarker cascade leading to cognitive impairment. First, we analyzed the interaction of sex and centiloid (independent variables) on tau SUVR in the meta-ROI and on GFAP as dependent variables in two separate models. Next, we separately analyzed the interaction of sex and tau SUVR in the meta-ROI (independent variables) on neurodegeneration (dependent variable) (measured as hippocampal volume, AD cortical thickness, and early-phase tau SUVR) and on neuroinflammation (dependent variable) (as measured by blood GFAP). Then, we explored possible interaction effects of sex and GFAP (independent variable) on neurodegeneration (dependent variable) (measured as hippocampal volume, AD cortical thickness, and early-phase tau SUVR).

Then, we assessed the associations of each biomarker and cognitive performance and decline, including related sex moderation effects. Specifically. we separately analyzed the interaction of sex with amyloid, tau SUVR in the meta-ROI, GFAP, and neurodegeneration (measured as hippocampal volume, AD cortical thickness, and early-phase tau SUVR) as independent variables on cognition measured at baseline as MMSE and over time as MMSE rate of changes as dependent variables. We also assessed the interaction effects between sex and non-AD specific biomarkers, namely those for white matter lesions (ARWMC and TLV) as independent variables on MMSE and MMSE rate of changes as dependent variables.

Sex differences in cognitive trajectories were assessed using separate longitudinal linear mixed-effect models with random intercepts and slopes using longitudinal MMSE as a dependent variable adjusting for age and each biomarker and sex as the predictor. These longitudinal models include a time × sex × biomarker interaction term (three-way interaction) to evaluate whether sex interacted with biomarker level in association with change in MMSE over the follow-up period.

Sensitivity analyses were run to examine the effect of removing individuals with negative amyloid-PET scans (amyloid positive subsample, *n* = 161), and to examine findings in only prodromal individuals (*n* = 193), including only CU and MCI.

All analyses were performed using R, version 4.0.2 (https://www.r-project.org/). A p-value of 0.05 was considered the significance threshold for all analyses and no correction for multiple comparisons was performed.

### Data availability

The data that support the findings of this study are available from the corresponding author, upon reasonable request.

## Results

**Fig. 1:**
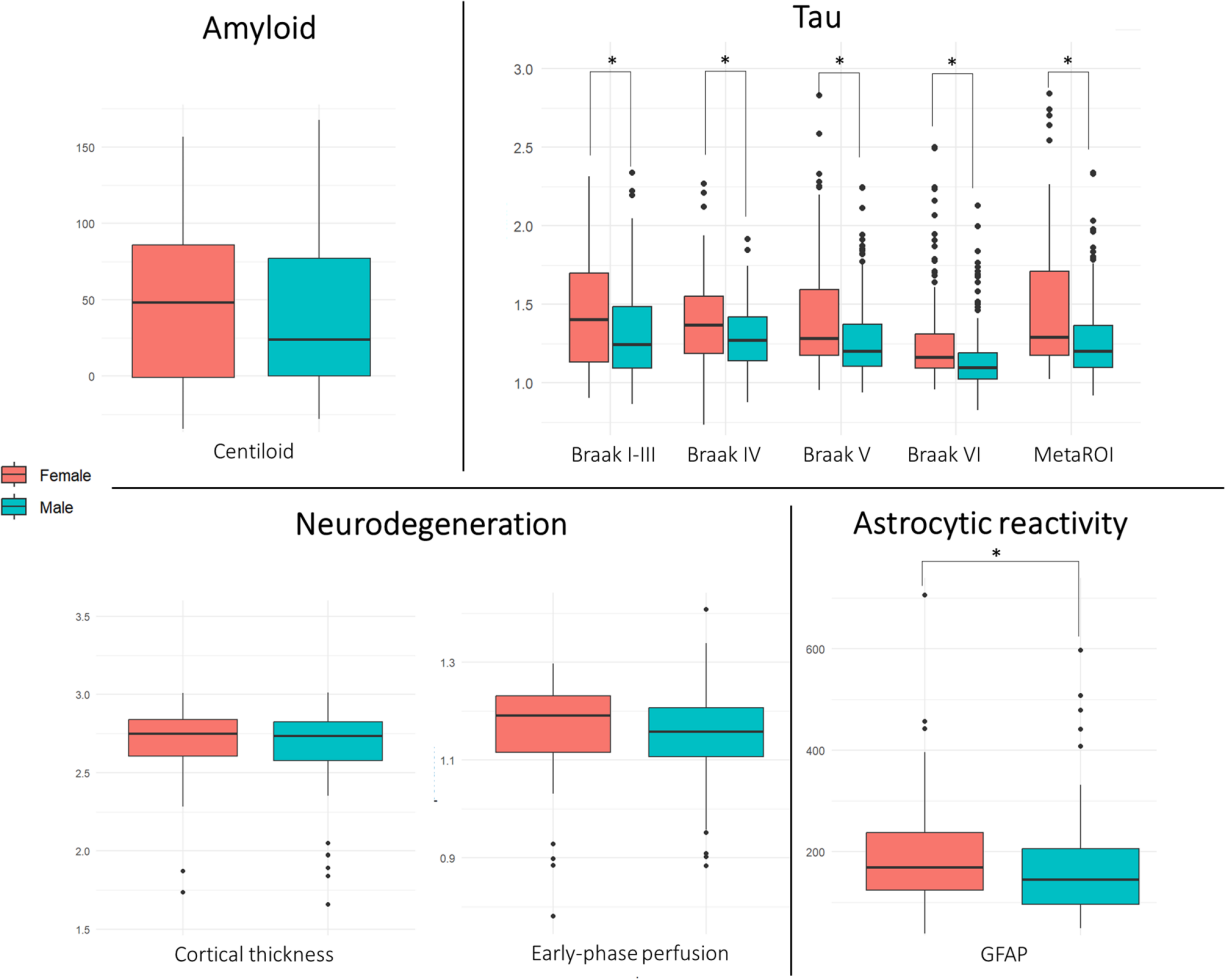
Sex-stratified box plots of Centiloid, tau SUVR in different regions, AD cortical thickness, early-phase perfusion SUVR in AD-related metaROI, and GFAP levels. The asterisk indicates significant sex differences (*p* < 0.05)

Clinical and demographic features of the whole group (females/males: 123/126) are reported in Table 2. The mean age was age 69.5 ± 9 and the majority of subjects were MCI. Female and male patients presented similar clinical and demographic features, amyloid, neurodegeneration (measured by MRI hippocampal volume, cortical thickness, and tau-PET early-phase hypoperfusion), and white matter changes (as measured by ARWMC and TLV). Females had a higher tau SUVR in all considered regions than males (*p* < 0.001) (Fig. 1), also confirmed by the sensitivity analyses and when controlling for other biomarkers. Females showed significantly higher GFAP levels than males (Fig. 1), but this difference did not persist when controlling for the other biomarkers.

**Table 2:** Demographic, clinical, and biomarker features of the whole sample

	Females	Males	*p*-value
	*N* = 123	*N* = 126	
**Clinical features**			
Age (years)	69.2 (9.7)	69.9 (8.5)	0.577
Education (years)	13 (3.9)	14 (4.3)	0.133
Caucasian ethnicity (%)	86%	93%	0.465
Clinical stage (CU/MCI/DEM)	37/57/29	29/70/27	0.311
MMSE	25.2 (4.2)	25.3 (4.9)	0.882
*APOE* genotype (ε2/ε3 / ε3/ε3 / ε3/ε4 / ε4/ε4)	11/32/30/5 14%/41%/38.5%/6.5%	9/41/21/3 12%/56%/28%/4%	0.313
Amyloid positivity (%)	59%	58%	0.980
**Biomarkers**			
Hippocampal volume (relative)	0.0025 (± 0.00040)	0.0025 (± 0.00038)	0.818
AD cortical thickness (mm)	2.70 (0.19)	2.68 (0.24)	0.452
Early-phase tau SUVR	1.30 (0.12)	1.28 (0.15)	0.313
Centiloid	45.6 (48.3)	40.2 (48.0)	0.442
Meta-ROI tau SUVR	1.47 (0.40)	1.29 (0.27)	**<0.001**
Braak I-III tau SUVR	1.45 (0.34)	1.32 (0.31)	**0.002**
Braak IV tau SUVR	1.39 (0.26)	1.30 (0.21)	**0.001**
Braak V tau SUVR	1.42 (0.36)	1.29 (0.27)	**0.001**
Braak VI tau SUVR	1.29 (0.36)	1.16 (0.23)	**0.001**
GFAP (pg/ml)	200 (110)	170 (110)	**0.044**
ARWMC	7.3 (± 5.1)	6.5 (± 5.0)	0.315
TLV (mm^3^)	4.5 (± 6.6)	5.9 (± 11)	0.506

Note: Continuous variables are reported as mean and standard deviation in the parenthesis, categorical variables as number and percentage in the parenthesis. All p-values are obtained by Mann-Whitney U tests for continuous variables and proportion test for frequencies

APOE genotyping was available for a subgroup of 152 participants; amyloid positivity based on PET for 186 participants; and GFAP for 135 participants

Abbreviations: AD, Alzheimer’s disease; ARWMC, age-related white matter change scale; CU, cognitively unimpaired; DEM, dementia; MCI, mild cognitive impairment; MMSE, mini-mental state examination; GFAP, Glial Fibrillary Acidic Protein; ROI, region of interest; SUVR, standardized uptake value ratios; TLV, total lesion volume

### Sex moderation effects on the associations between AD biomarkers

**Fig. 2:**
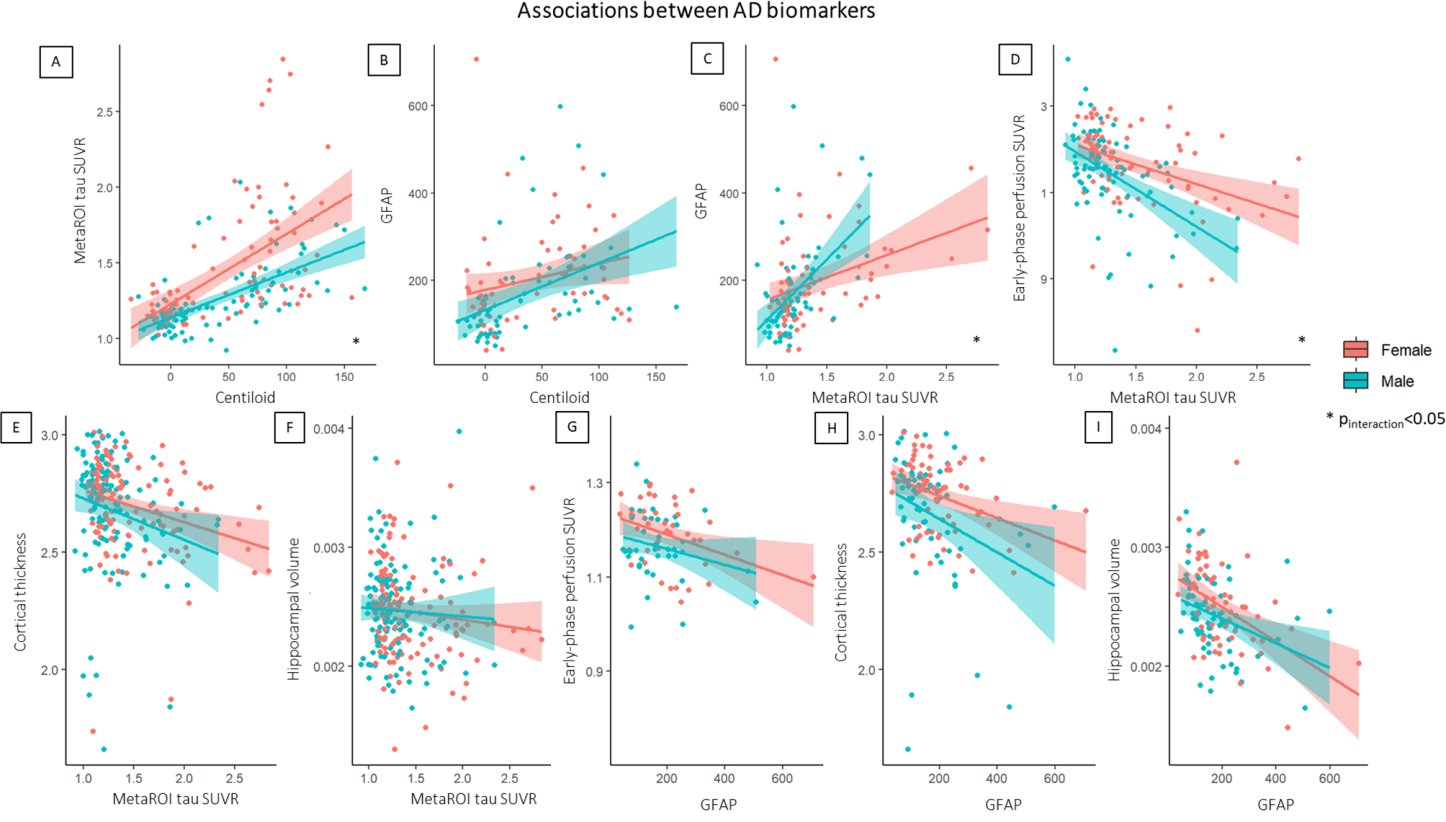
Associations between AD biomarkers in females and males. Linear regressions show the different associations between amyloid (centiloid), tau (SUVR in AD metaROI), neurodegeneration (early-phase perfusion SUVR in AD-related metaROI, cortical thickness, hippocampal volume) and GFAP levels. The figure reports all associations and those characterized by a significant moderation effect by sex are marked by * indicating *p* < 0.05. The shaded areas around each regression line in the plots represent the confidence intervals for the regression lines

We found a significant association between amyloid centiloid and tau SUVR in the meta-ROI (standardized β = 0.54, *p* < 0.001), with a significant sex-by-amyloid interaction indicating a stronger association among women than men in the whole sample (standardized β=-0.19, *p* = 0.047) (Fig. 2A). The association between tau SUVR and neurodegeneration was significant using both AD cortical thickness (standardized β=-0.24, *p* = 0.005) and early-phase perfusion (standardized β=-0.44, *p* < 0.001), but not the hippocampal volume; however, a significant sex-by-tau interaction effect was found only on early-phase perfusion indicating a stronger association in men in the whole sample (standardized β=-0.56, *p* = 0.044) (Fig. 2D) and in amyloid-positive subsample (standardized β=-1.54, *p* = 0.012). Linear regression showed a significant association between tau SUVR in the meta-ROI and GFAP (standardized β = 0.40, *p* < 0.001) with a significant sex-by-tau interaction effect indicating a stronger association in men (standardized β = 0.99, *p* = 0.013) (Fig. 2C). We didn’t find any significant sex moderation effect on the relationship between amyloid centiloid and GFAP in the whole group (Fig. 2B), even if the association was significant only in males (standardized β = 1, *p* < 0.001) when considered separately. Also, the associations between GFAP and neurodegeneration measures were significant (hippocampal volume: standardized β=-0.40, *p* < 0.001, cortical thickness: standardized β=-0.31, *p* = 0.001; early-phase: standardized β=-0.30, *p* = 0.013) but without significant moderation effects by sex (Fig. 2G, H, I). Regarding non-AD specific biomarkers, we did not find any significant associations between white matter lesion biomarkers (ARWMC and TLV) and AD biomarkers, except for the hippocampal volume (standardized β=-0.201, *p* = 0.003) but without significant sex moderation effects.

### Sex moderation effects on the associations between biomarkers and cognitive performance and decline

**Fig. 3:**
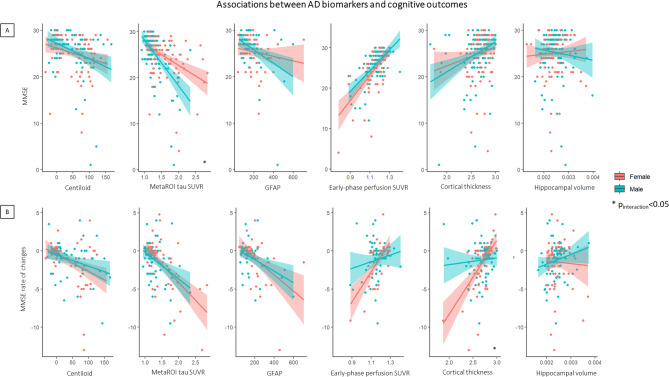
Associations between AD biomarkers and cognitive outcomes in females and males. Linear regressions show the different associations between amyloid (centiloid), tau (SUVR in AD metaROI), neurodegeneration (early-phase perfusion SUVR in AD-related metaROI, cortical thickness, hippocampal volume) and GFAP levels and cognitive performance (MMSE at baseline, **A**) and decline (MMSE rate of changes, **B**). The figure reports all associations and those characterized by a significant moderation effect by sex are marked by * indicating *p* < 0.05. The shaded areas around each regression line in the plots represent the confidence intervals for the regression lines

Linear regression showed a significant effect of tau SUVR in the meta-ROI on MMSE at baseline (standardized β=-0.48, *p* < 0.001) with a significant sex-by-tau interaction effect indicating a stronger association among men than women in the whole group (standardized β=-0.76, *p* = 0.001) (Fig. 3A), and in the amyloid-positive subsample (standardized β=-1.21, *p* = 0.017). Hippocampal volume was not associated with MMSE at baseline (*p* = 0.785). Despite a significant association between early-phase tau and MMSE (standardized β = 0.61, *p* < 0.001), and AD cortical thickness and MMSE (standardized β = 0.25, *p* < 0.001), they were not moderated by a significant interaction by sex (Fig. 3A). No sex moderation effect was found on the relationship between GFAP and MMSE (Fig. 3A), even if the association was significant only in males (standardized β=-0.33, *p* = 0.002) when considered separately. Regarding non-AD specific biomarkers, we did not find any significant associations between white matter lesion biomarkers (ARWMC and TLV) and MMSE (*p* > 0.05, Figure S2).

Although all biomarkers, except for hippocampal volume and white matter lesions, correlated with cognitive decline in terms of MMSE rate of changes (amyloid: standardized β=-0.35, *p* < 0.001; tau: standardized β=-0.52, *p* < 0.001; early-phase: standardized β = 0.35, *p* = 0.013, cortical thickness: standardized β = 0.34, *p* = 0.006; GFAP: standardized β=-0.48, *p* < 0.001), a significant moderation effect with a stronger association in women than men was found only with cortical thickness as a measure of neurodegeneration in the whole sample (standardized β=-4.35, *p* = 0.001) (Fig. 3B) and the prodromal sample (standardized β=-5.05, *p* = 0.003). The same result was confirmed by longitudinal linear mixed-effect models using longitudinal MMSE as a dependent variable; we found a significant three-way interaction effect showing females and males with different cognitive trajectories depending on the cortical thinning severity (standardized β=-0.34, *p* < 0.001). Females with high atrophy (lower tertile) exhibited faster cognitive decline than men with high atrophy and males with low atrophy (upper tertile) exhibited faster cognitive decline then females with low atrophy (Figure S3). No significant three-way interaction effects were found with the other biomarkers, namely Centiloid (standardized β = 0.09, *p* = 0.56), tau SUVR in the metaROI (standardized β=-0.33, *p* = 0.49), blood GFAP (standardized β = 0.032, *p* = 0.20), early-phase perfusion SUVR (standardized β=-1.58, *p* = 0.52), hippocampal volume (standardized β=-0.22, *p* = 0.82), and white matter lesions (ARWMC: standardized β=-0.19, *p* = 0.45; TVL: standardized β = 0.02, *p* = 0.92).

## Discussion

The mechanisms underlying AD aetiology and progression are complex and multifactorial and sex is an important factor in phenotypic and endophenotypic variability. This study provided the first evaluation of the influence of sex on the associations between AD-related pathological alterations, considering amyloid, tau, neurodegeneration, neuroinflammation, and white matter lesions as well as cognitive measures. Despite clinical comparability between the two sexes, females showed higher tau loads and a trend for higher plasma GFAP levels than males. Moreover, the sex difference in tau burden was exacerbated in the setting of high neocortical amyloid. On the other hand, in individuals with high tau burden, males exhibited greater neurodegeneration as measured by hypoperfusion and lower cognitive performance than females. Despite the absence of sex differences in cognitive trajectories per se, in the presence of severe cortical thinning, females exhibited faster cognitive decline than males. Our results suggest that women may have greater reserve, against their greater tau burden, allowing them to cope better with neurodegeneration and cognitive impairment at the beginning, but when they reach a certain threshold women’s cognitive decline is faster than men’s. Reserve may protect women at the prodromal phases, but later, when they reach high levels of neurodegeneration, they exhibited steeper cognitive decline than men. Previous studies reported women with MCI showing greater cognitive decline than men [32] and also that women progressed faster than men to a clinical diagnosis of MCI and dementia [33–35]. However, we cannot exclude that the faster cognitive decline observed in women could result from later diagnosis of AD in women than men [7].

The result of greater tau loads in females is in line with recent studies reporting higher tau in medial temporal regions in cognitively unimpaired individuals, and also in temporoparietal regions in individuals with MCI and dementia [9–16]. In this memory clinic cohort, we found higher tau loads in AD-related regions including all Braak stages in females compared to males. These differences remained significant even when controlling for other biomarkers, clinical stages, and age. Sex disparities in protein degradation pathways, particularly autophagy that is lower in women than in men throughout life, along with chromosomal and hormonal factors have been proposed as possible mechanistic underpinnings of differences in tau [36]. Earlier age at menopause and the late initiation of hormonal replacement therapy following menopause onset have been associated with increased tau vulnerability in cognitively unimpaired females [37]. Moreover, these previous results were exacerbated in individuals with high amyloid pathology [9, 37], according to the fact that reduced autophagic induction or flux results in a failure to clear protein aggregates, and this aggregation further inhibits autophagy, resulting in self-sustaining pathology [36]. Accordingly, we found a significant moderation effect of sex on the association between amyloid and neocortical tau indicating a stronger association in females than males leading to higher tau in the presence of high amyloid burden. A similar result has been found in clinically normal women who exhibited higher tau in the entorhinal cortex than men in individuals with high amyloid burden [9]. A secondary pathway driven by sex-specific lifestyle determinants, such as inflammation that is heightened in women, has been proposed to partially explain the sex-modifying effect on the association between amyloid and tau [9]. However, we did not find a sex moderation effect on the association between amyloid and inflammation as measured by GFAP in our cohort, and sex differences in GFAP did not survive the corrections for other biomarkers. Glia sex differences have been documented in several innate immune responses and neuroinflammatory phenotypes [3] with female and male astrocytes differing in their secretion of trophic factors, neuroinflammatory molecules, and neuroactive steroids, in their metabolic supply to neurons and in their control of the neurovascular unit and the blood-brain barrier [38]. However, the immune genes on the X chromosome-linked to inflammation are in complex relationships with oestrogen and aging and thus may not necessarily translate to higher global inflammation in women [3].

Despite the greater burden of pathology in females, in the presence of high tau males showed greater neurodegeneration and astrocyte reactivity and lower cognitive performance than females as shown by significant tau-by-sex interactions on perfusion, GFAP, and MMSE measures, respectively. Perfusion is closely related to cerebral glucose metabolism, a gold standard measure of neurodegeneration, based on neurovascular coupling [39], and there are many evidence supporting its use as a surrogate biomarker of neural injury as well [30, 40]. These results suggest that females may hold a greater tau burden without manifesting severe neurodegeneration as well as cognitive deficits compared to males. A greater brain resilience to pathological tau in terms of relative preservation of brain structure when exposed to neocortical tau has been proven in vivo in women with AD compared to men [41]. Females’ higher premorbid abilities in verbal memory have been advanced as a possible explanation for the better handling of brain pathology that could act to buffer the effects of neurodegeneration with time-limited advantages [42]. Previous studies have found greater cognitive resilience to AD pathology in females with AD [43, 44] and among cognitively unimpaired individuals at genetic risk for autosomal-dominant AD [45]. Similarly, a more pronounced association between cognitive activities and cognitive reserve in women than in men has been documented, suggesting greater beneficial effects of lifestyle activities on cognitive reserve in women than men [46]. However, females’ advantages may not equate to better performance over the entire disease course given the evidence of faster cognitive decline and tau accumulation rates in women with MCI and dementia [7, 17, 43], but also in cohorts of cognitively normal adults [44, 47]. In line with previous evidence, our longitudinal results showed faster cognitive decline in females than men but only in the presence of high atrophy as demonstrated by a significant three-way interaction effect. Overall, this result suggests that even if females may take longer to reach a high neurodegeneration burden in the presence of high tau, when they do, their cognitive decline is faster than in men.

Limitations of the present study include first the incapability to distinguish between gender constructs and biological sex. In fact, beyond sex-related factors, social influences experienced especially by old people might affect the progression and outcome of neurodegenerative diseases [48]. Moreover, given the importance of sex differences in the burden and manifestations of cardiovascular risk factors [7], their impact on AD deserves to be systematically investigated. Second, we used MMSE as a measure of cognitive decline, although we are aware that MMSE is a global measure characterized by a ceiling effect. Other tests or test combinations should be tested as more sensitive [49, 50]. Third, we evaluated our subjects with a relatively short follow-up period. We regard the heterogeneity of our sample from the memory clinic as a strength of the study, enhancing the relevance of our results to clinical settings. However, we admit that the generalizability of our results is restricted to memory clinics excluding community dwellers, and to the Caucasian community, since other ethnic groups are underrepresented here. While our memory clinic sample was predominantly composed of individuals with AD, and our findings are likely influenced by these subjects, we recognize that some cases might involve non-AD pathologies and for this reason, we have run sensitivity analyses.

## Conclusions

These findings support the notion that sex differences may affect tau deposition, driven by differences in the upstream interplay with amyloid, and lead to different downstream effects on neurodegeneration and cognitive outcomes. Given the multifaceted sex differences observed in biomarkers and their associations as well as in clinical progression, it remains highly likely that sex will need to be considered to move the field towards more precise and effective treatment strategies.

## Electronic supplementary material

Below is the link to the electronic supplementary material.

Supplementary Material 1

## Data Availability

Anonymized data used in this study are available upon reasonable request from the corresponding author (VG).
