# Supplementary material for: Sex differences in the association of Alzheimer’s disease biomarkers and cognition in a multicenter memory clinic study
Source: Alzheimers Res Ther. 2025 Feb 18;17:46. doi: 10.1186/s13195-025-01684-z (PMC11837373; doi:10.1186/s13195-025-01684-z)

**Supplementary Materials**

**Table S1.** Demographic, clinical, and biomarker features of the subsample with available GFAP measurement

|  | **Females** | **Males** | **p-value** |
| --- | --- | --- | --- |
|  | N=67 | N=68 |  |
| **Clinical features** |  |  |  |
| Age (years) | 69.1 (10.2) | 71.3 (8.68) | 0.196 |
| Education (years) | 13.5 (4.33) | 14.8 (3.98) | 0.066 |
| Caucasian ethnicity | 82% | 90% | 0.487 |
| Clinical stage (CU/MCI/DEM) | 24/35/8 | 17/43/8 | 0.345 |
| MMSE | 25.3 (3.46) | 26.2 (4.35) | 0.268 |
| APOE ε4 carriership (%) | 44.6% | 30.8% | 0.148 |
| Amyloid positivity (%) | 54% | 52.4% | 1 |
| **Biomarkers** |  |  |  |
| Hippocampal volume (relative) | 0.0025 (± 0.00040) | 0.0025 (± 0.00038) | 0.252 |
| AD cortical thickness (mm) | 2.74 (0.15) | 2.66 (0.27) | **0.040** |
| Early-phase Tau SUVR | 1.31 (0.10) | 1.29 (0.12) | 0.285 |
| Centiloid | 39.8 (44.3) | 38.3 (44.3) | 0.844 |
| Global Tau SUVR | 1.43 (0.39) | 1.23 (0.20) | **<0.001** |
| Braak I-III Tau SUVR | 1.39 (0.36) | 1.29 (0.29) | 0.079 |
| Braak IV Tau SUVR | 1.38 (0.26) | 1.28 (0.21) | **0.017** |
| Braak V Tau SUVR | 1.38 (0.33) | 1.24 (0.22) | **0.006** |
| Braak VI Tau SUVR | 1.25 (0.30) | 1.13 (0.19) | **0.006** |
| GFAP (pg/ml) | 200 (110) | 170 (110) | **0.044** |
| ARWMC | 7.73 (5.62) | 7.31 (5.23) | 0.713 |
| TLV (mm3) | 4.74 (7.77) | 7.22 (12.8) | 0.190 |
| Note: Continuous variables are reported as mean and standard deviation, categorical variables as number and percentage. All p-values are obtained by  Mann-Whitney U tests for continuous variables and proportion test for frequencies.  Abbreviations: AD, Alzheimer’s disease; ARWMC, age-related white matter change scale; CU, cognitively unimpaired; DEM, dementia; MCI, mild cognitive impairment; MMSE, mini-mental state examination; GFAP, Glial Fibrillary Acidic Protein; SUVR, standardized uptake value ratios; TLV, total lesion volume. | | | |

**Table S2.** Demographic, clinical, and biomarker features of the subsample with available clinical follow-up

|  | **Females** | **Males** | **p-value** |
| --- | --- | --- | --- |
|  | N=68 | N=69 |  |
| **Clinical features** |  |  |  |
| Age (years) | 71.4 (7.48) | 71.2 (6.97) | 0.931 |
| Education (years) | 13.3 (3.99) | 14.8 (4.23) | **0.046** |
| Caucasian ethnicity | 86% | 93% | 0.624 |
| Clinical stage (CU/MCI/DEM) | 27/30/11 | 19/43/7 | 0.101 |
| MMSE | 25.7 (3.18) | 25.6 (4.96) | 0.861 |
| APOE ε4 carriership (%) | 46.2% | 32.7% | 0.148 |
| Amyloid positivity (%) | 52.3% | 51.5% | 1 |
| **Biomarkers** |  |  |  |
| Hippocampal volume (relative) | 0.0025 (± 0.00040) | 0.0025 (± 0.00038) | 0.229 |
| AD cortical thickness (mm) | 2.70 (0.18) | 2.67 (0.23) | 0.438 |
| Early-phase Tau SUVR | 1.31 (0.10) | 1.30 (0.13) | 0.688 |
| Centiloid | 39.6 (50.0) | 38.9 (50.2) | 0.941 |
| Global Tau SUVR | 1.43 (0.39) | 1.30 (0.28) | **0.026** |
| Braak I-III Tau SUVR | 1.42 (0.34) | 1.35 (0.35) | 0.233 |
| Braak IV Tau SUVR | 1.37 (0.24) | 1.30 (0.22) | 0.070 |
| Braak V Tau SUVR | 1.38 (0.32) | 1.31 (0.30) | 0.205 |
| Braak VI Tau SUVR | 1.24 (0.25) | 1.17 (0.25) | 0.146 |
| GFAP (pg/ml) | 203 (119) | 170 (124) | 0.204 |
| ARWMC | 8.16 (5.80) | 7.32 (5.08) | 0.472 |
| TLV (mm3) | 5.30 (7.62) | 6.60 (9.44) | 0.407 |
| Note: Continuous variables are reported as mean and standard deviation, categorical variables as number and percentage. All p-values are obtained by  Mann-Whitney U tests for continuous variables and proportion test for frequencies.  Abbreviations: AD, Alzheimer’s disease; ARWMC, age-related white matter change scale; CU, cognitively unimpaired; DEM, dementia; MCI, mild cognitive impairment; MMSE, mini-mental state examination; GFAP, Glial Fibrillary Acidic Protein; SUVR, standardized uptake value ratios; TLV, total lesion volume. | | | |

**Figure S1. Meta ROI for early-phase perfusion images.** A voxel-wise linear regression model including intensity-normalized early-phase tau images and the MMSE scores was used to identify a relevant meta-ROI for early-phase perfusion. The statistical threshold was set at p<0.005, FWE-corrected at the cluster level. The obtained ROI resembled the Alzheimer’s disease-typical hypometabolic pattern including temporoparietal and frontal regions and has been used to extract the SUVR for early-phase perfusion images.


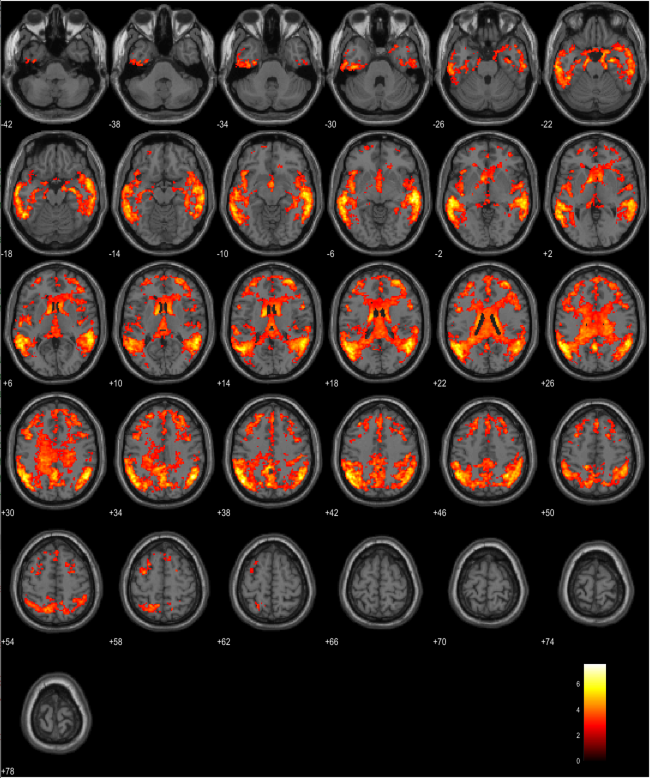


**Figure S2. Associations between white matter lesions and cognitive outcomes in females (pink) and males (blue).** Linear regressions show the different associations between ARWMC and TLV and cognitive performance (MMSE at baseline) and decline (MMSE rate of changes). The shaded areas around each regression line in the plots represent the confidence intervals for the regression lines.


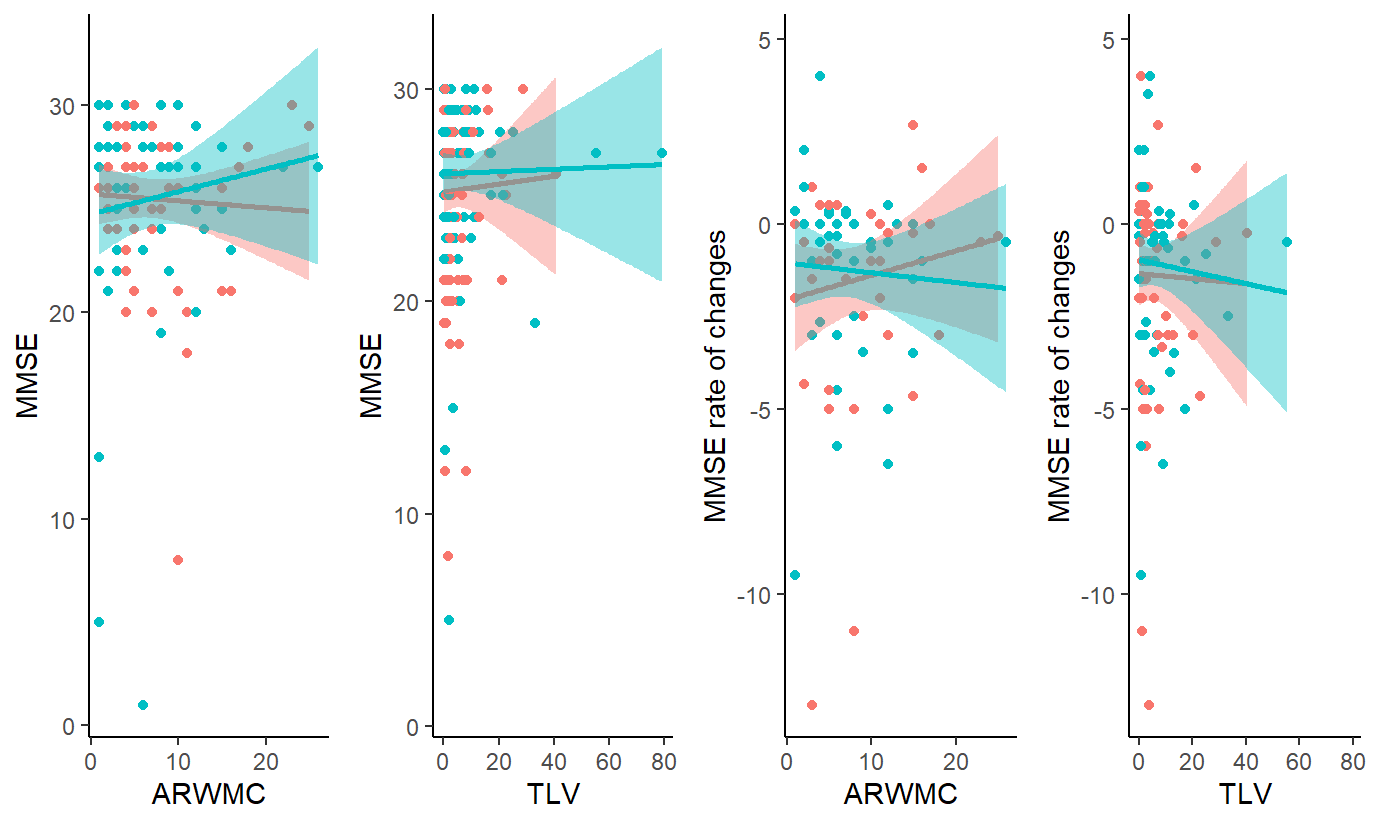


**Figure S3. Longitudinal results show different cognitive trajectories of MMSE scores over time in females and males at different levels of AD cortical thickness.** Results obtained with the longitudinal linear mixed-effect model showed a significant three-way interaction effect showing that females with high atrophy (lower tertile) exhibited faster cognitive decline than men with high atrophy, and males with low atrophy (upper tertile) exhibited faster cognitive decline then females with low atrophy.


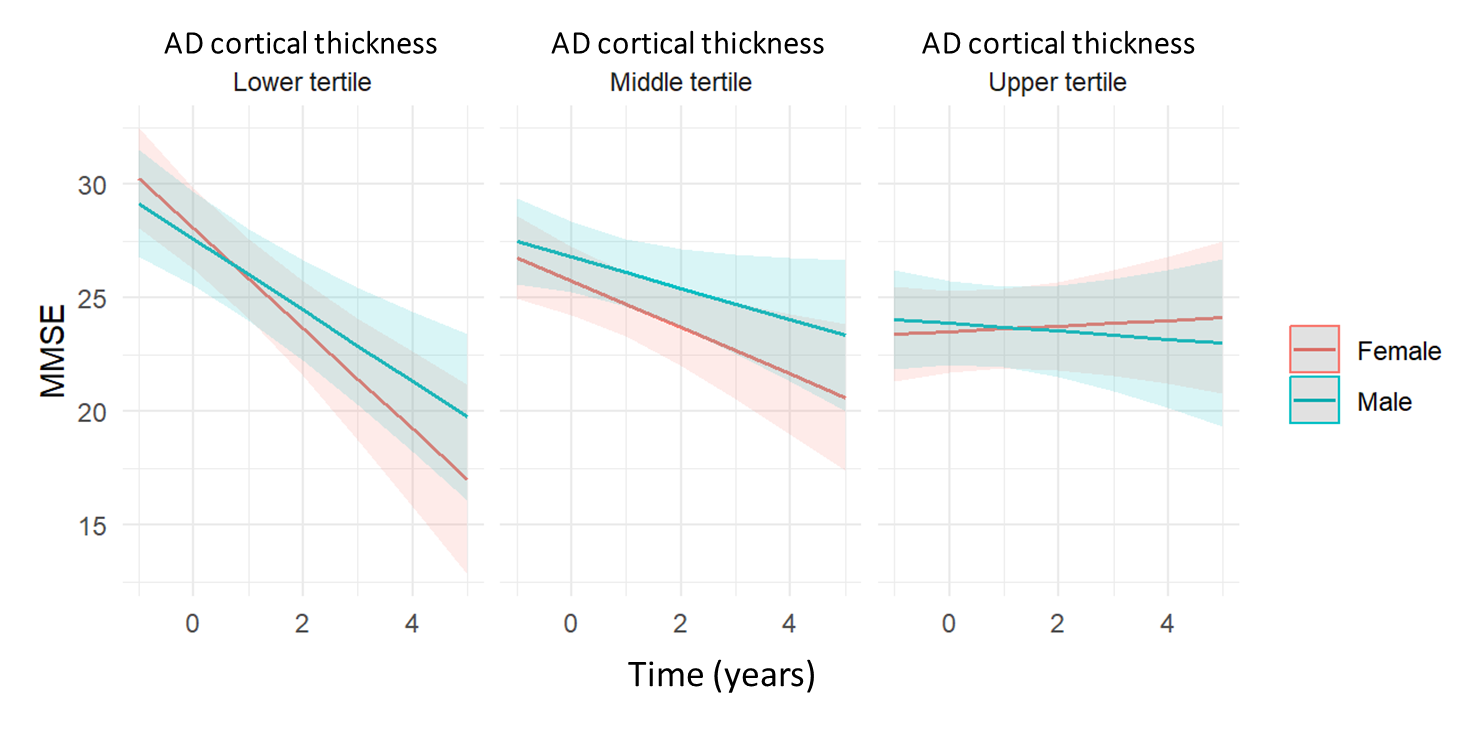

Supplement: Supplementary file 1 — Supplementary Material 1 [file 13195_2025_1684_MOESM1_ESM.docx]
